# Exercise‐induced bronchoconstriction assessed by a ratio of surface diaphragm EMG to tidal volume

**DOI:** 10.14814/phy2.15860

**Published:** 2023-11-13

**Authors:** Lishuang Wang, Senrui Wu, Baiting He, Simin Liu, Shanfeng Liang, Yuanming Luo

**Affiliations:** ^1^ State Key Laboratory of Respiratory Disease Guangzhou Medical University Guangzhou China; ^2^ Division of Sleep and Circadian Disorders Brigham and Women's Hospital and Harvard Medical School Boston Massachusetts USA; ^3^ College of Medicine and Public Health, Adelaide Institute for Sleep Health Flinders University Adelaide South Australia Australia

**Keywords:** airway hyperresponsiveness, asthma, diaphragm electromyogram, exercise‐induced bronchoconstriction

## Abstract

Exercise‐induced bronchoconstriction (EIB) is usually assessed by changes in forced expiratory volume in 1 s (FEV_1_) which is effort dependent. The purpose of this study was to determine whether the diaphragm electromyogram (EMG_di_) recorded from chest wall surface electrodes could be used to reflect changes in airway resistance during an exercise challenge test and to distinguish patients with EIB from those without EIB. Ninety participants with or without asthma history were included in the study. FEV_1_ was recorded before and 5, 10, 15, and 20 min after exercise. EIB was defined as an FEV_1_ decline greater than 10% after exercise. A ratio of root mean square of EMG_di_ to tidal volume (EMG_di_/*V*_T_) was used to assess changes in airway resistance. Based on changes in FEV_1_, 25 of 90 participants exhibited EIB; the remainder were defined as non‐EIB participants. EMG_di_/*V*_T_ in EIB increased by 124% (19%–478%) which was significantly higher than that of 21% (−39% to 134%) in non‐EIB participants (*p* < 0.001). At the optimal cutoff point (54% in EMG_di_/*V*_T_), the area under the ROC curve (AUC) for detection of a positive test was 0.92 (*p* < 0.001) with sensitivity 92% and specificity 88%. EMG_di_/*V*_T_ can be used to assess changes in airway resistance after exercise and could be used to distinguish participants with EIB from those without EIB.

## 1 INTRODUCTION

Exercise‐induced bronchoconstriction (EIB) refers to a temporary and acute contraction of the bronchioles in patients with or without asthma during or after exercise (Randolph, 2009). EIB is common with a prevalence of 5%–20% in the general population, 30%–70% in elite athletes and up to 35% in children under 16 years old (Boulet & O'Byrne, 2015; Cichalewski et al., 2015; Helenius & Haahtela, 2000; Johansson et al., 2015; Price, Sewry, et al., 2022). Furthermore, the incidence of EIB in people with chronic asthma, especially among those poorly controlled, can be as high as 90% (Weiler et al., 2007). The pathogenesis of EIB is mainly related to sustained high minute ventilation, augmented osmolarity of airway surface liquid and release of inflammatory mediators, leading to contraction of bronchial smooth muscles and an increase in airway resistance which in turn impairs the patient's exercise endurance and quality of life (Anderson & Kippelen, 2005; Hallstrand et al., 2005). Cough, wheezing, or shortness of breath are commonly reported by EIB patients but not specific to EIB, causing a delay in diagnosis. Repeated occurrences of EIB may increase patients' psychological and financial burdens (Aggarwal et al., 2018; Parsons & Mastronarde, 2009; Weiler et al., 2016).

EIB is usually diagnosed by exercise challenge test although an indirect bronchial provocation test (e.g., eucapnic voluntary hyperpnea or inhaled mannitol) can also be used in clinical practice (Price, Walsted, et al., 2022). A decrease of forced expiratory volume in 1 s (FEV_1_) greater than 10% after exercise is considered diagnostic of EIB according to guidelines (Parsons & Mastronarde, 2009). However, accurate measurements of FEV_1_ depend on the cooperation between operators and participants who must consistently produce a maximal effort which could be difficult for those with learning difficulties or children (Crapo, 1994).

It is hypothesized that diaphragm EMG increases with narrowing in the lower airway providing that the rest of respiratory mechanics is the same. The diaphragm electromyogram (EMG_di_) recorded from chest wall surface electrodes is a noninvasive technique which has been used to reflect changes in lower airway resistance induced by the histamine provocation test (Maarsingh et al., 1985, 2002, 2004). Because EMG_di_ changes with volitional effort, we hypothesized that the ratio of surface EMG_di_ to tidal volume (EMG_di_/*V*_T_) could be more useful than an increase in EMG_di_ alone in distinguishing participants with EIB from those without EIB.

## 2 METHODS

### 2.1 Subject recruitment

A total of 98 participants were recruited for the study, of which 17 were healthy laboratory staff and the rest were patients with suspected asthma from outpatient clinics of the First Affiliated Hospital of Guangzhou Medical University. Participants with severe heart disease, anemia, and neuromuscular disease were excluded. Participants were asked to stop all inhaled bronchodilators or corticosteroids for 24 h before the test. Written informed consent was obtained from all subjects. The study was approved by the ethics committee of the First Affiliated Hospital of Guangzhou Medical University (Medical Research Ethics review: 2020K‐123).

### 2.2 Lung function test and exercise challenge test

All subjects performed spirometry (Cosmed Micro Quark, Cosmed Italy) based on the recommendation of ATS/ERS (Crapo et al., 2000; Hallstrand et al., 2018). Sufficient time was provided to all the participants for practicing maneuvers of the forced spirometry to ensure the quality of the pulmonary function tests. At least three maximal repeatable tests were obtained before the formal test. Spirometry was performed before exercise and 5, 10, 15, 20 min after exercise, and a minimum of two spirometry maneuvers were performed for each time point. The largest FEV_1_ value at each time point was used for further analysis.

Treadmill (Polar cos10253‐01 made in Germany) exercise was performed. Participants were required to reach a target heart rate (85% of maximal heart rate (220‐age) within 2–4 min and maintain it for 6 min, with a target of the total exercise duration not exceeding 15 min. Accordingly, the treadmill speed was increased rapidly to the participants' maximal bearable level and then raised gradient to further increase the exercise intensity until the target heart rate was achieved. If the participants developed intolerant symptoms such as dyspnea and dizziness or the oxygen saturation fell below 90%, exercise was terminated. Salbutamol inhalation of 200 μg was offered to participants who experienced severe breathing difficulty associated with a significant reduction in FEV_1_ (>10% from baseline) which had not recovered at 20 min after exercise.

### 2.3 EMG_di_ and *V*_T_ recording

EMG_di_ was simultaneously recorded from two pairs of electrodes (3M Health Care, D‐41453 Neuss, Germany). In Pair 1, electrodes were positioned at the tip of the xiphoid process and the right costal margin with a distance of 16 cm between two electrodes (Chen et al., 1995); in Pair 2, electrodes were positioned at the sixth and eighth intercostal space along the left anterior axillary line (Luo et al., 1998). Respiratory airflow was recorded by a digital flow meter (Siargo, Inc. Santa Clara, CA 95054, USA) which was further connected to an additional tube (7 cm in length and 2 cm diameter) to increase inspiratory load to improve EMG_di_ quality. The EMG_di_ and respiratory airflow were recorded by the respiratory signal processor (RA‐16, Respiratory Medical Science Co. Ltd, Guangzhou, China) simultaneously. To facilitate comparison, all participants were required to breathe at the same respiratory rate of 15 times/min by following a sound pre‐recorded in the headphones. Three minutes' data were recorded while subjects sat upright with hands on the laps 15 min before exercise. EMG_di_ and tidal volume were recorded for 90 s immediately before spirometry at 3, 5, 10, 15, and 20 min, respectively, after exercise.

### 2.4 Data and statistical analysis

EMG_di_ was automatically converted to root mean square (RMS) by the respiratory signal processor (RA‐16, Respiratory Medical Science Co. Ltd, Guangzhou, China). Peak value of RMS of EMG free from ECG was recorded. Tidal volume was calculated from integrated flow and was further standardized according to body weight *V*_T_ (mL/kg). Participants were divided into the EIB group (FEV_1_ decreased ≥10% after exercise) and the control group (FEV_1_ decreased <10% after exercise). Data from 15 breathing cycles during tidal breaths were averaged for each time point (before exercise and 3, 5, 10, 15, and 20 min after exercise). The maximal changes of FEV_1_, EMG_di,_ and EMG_di_/*V*_T_ before and after exercise were selected for further analysis. EMG_di_ recorded from Pair 2 was regarded as the primary signal for analysis because it had less ECG artifacts. For each subject, the same pair of electrodes were used consistently before and after exercise. Changes in FEV_1_, EMG_di,_ and EMG_di_/*V*_T_ before and after exercise were expressed as ΔFEV_1_%, ΔEMG_di_%, and ΔEMG_di_%/*V*_T_, respectively.

All parameters were assessed for normality by the Shapiro–Wilk test. Normally distributed data were presented as mean ± SD while data distributed non‐normally were represented with mean (minimum–maximum). The mixed repeated ANOVA was applied for analysis in groups before and after exercise while *t*‐tests were also used when it was suitable. The results were considered to be statistically significant at *p* < 0.05. Spearman coefficients were calculated to determine the correlation between the decrease in FEV_1_ and the increase in EMG_di_ and EMG_di_/*V*_T_. ROC (receiver operating characteristic) curves were used to evaluate the diagnostic value of EMG_di_ and EMG_di_/*V*_T_ for EIB.

## 3 RESULTS

### 3.1 Characteristics of subjects

Ninety‐eight participants were studied with eight participants excluded from analysis because of poor electrode connection after sweating associated with exercise (5), leakage of air during tidal breathing (1), inability to follow the required breathing frequency (1), and failure in reaching the target heart rate during exercise (1). Finally, 90 participants (49 males) ranging from 10 to 66 years old were analyzed as shown in Table 1.

**TABLE 1:** The characteristic of EIB group and control group.

	Control group (*N* = 65)	EIB group (*N* = 25)	*p* value
Age (years)	36 (12–65)	43 (10–66)	0.064
M/F	34/31	15/10	0.512
Height (cm)	164.0 ± 7.9	157.3 ± 10.6	0.001
Weight (kg)	61.6 ± 11.1	59.7 ± 11.7	0.471
BMI	22.8 ± 3.2	24.0 ± 3.5	0.11
Ambient temperature (°C)	23.7 ± 1.4	23.0 ± 2.1	0.099
Ambient humidity (%)	63.1 ± 6.1	61.9 ± 5.6	0.545
FEV_1_ (L)	2.79 (1.12–5.04)	1.94 (0.86–3.83)	<0.001
FEV_1_ (% pred)	87 ± 17	71 ± 14	<0.001
FVC (L)	3.60 (1.63–6.34)	3.09 (1.78–4.92)	0.016
FVC (% pred)	95 ± 15	95 ± 12	0.962
FEV_1_/FVC (%)	78 ± 10	63 ± 11	<0.001

*Note*: Data are presented as mean ± SD or mean (minimum–maximum).

Abbreviations: BMI, body mass index; FEV_1_, forced expiratory volume in 1 s; % pred, % predicted; FVC, forced vital capacity.

**FIGURE 1:**
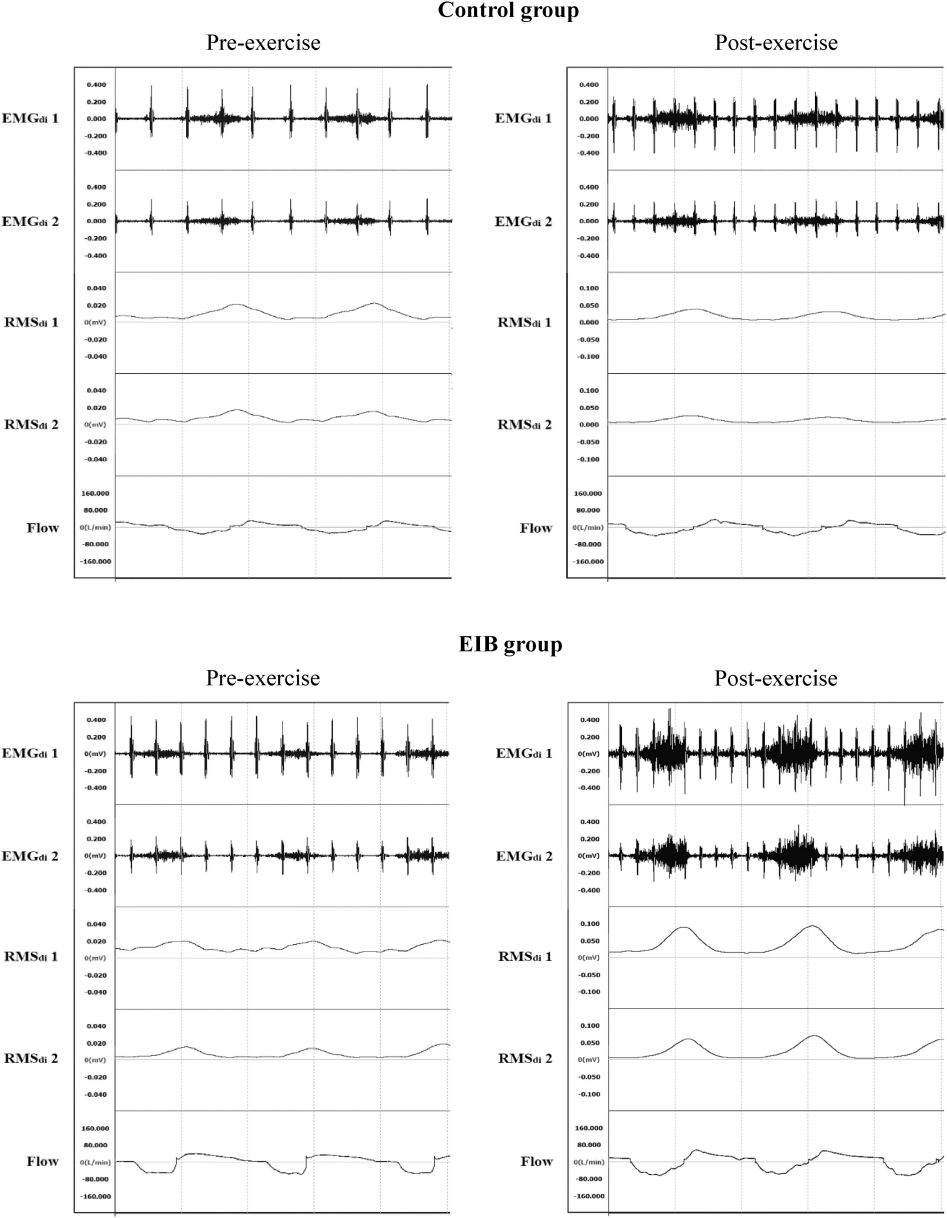
Comparison of EMG_di_ recordings between the control group and EIB group before and 3 min after exercise. EMG_di_1 and EMG_di_2 represent the EMG signals obtained from chest surface diaphragmatic electromyograms at electrode positions Pair 1 and Pair 2, respectively. RMS_di_1 and RMS_di_2 are the root mean square transformation of EMG_di_1 and EMG_di_2. Flow represents the airflow from breathing recorded by the electronic flow meter and is calibrated with inspiration as negative value. The scale of EMG_di_ is ±0.6 mV and the scale of RMS_di_ before exercise is 0.06 mV while the scale of RMS_di_ at 3 min after exercise is 0.15 mV.

**FIGURE 2:**
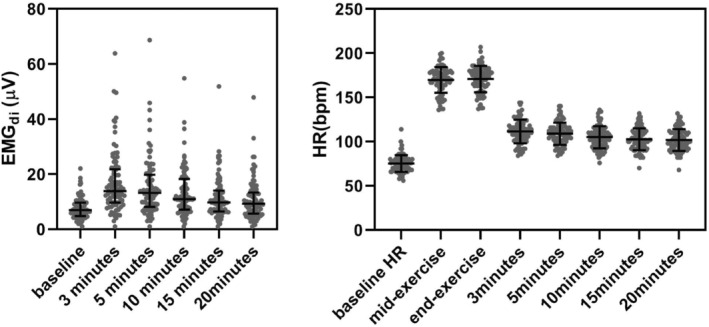
Scatter dot plots show the trend of EMG_di_ and heart rates (HR) during exercise as well as 3, 5, 10, 15, and 20 min after exercise. The EMG_di_ of all subjects presented a change of increasing first and then decreasing gradually (left). The error bar length is the interquartile range due to non‐normally distributed in EMG_di._ Moreover, all subjects could reach and maintain the target heart rates for a period of time, and heart rates dropped slowly from 3 to 20 min after exercise (right).

An example of EMG_di_ recording is shown in Figure 1. The baseline value of EMG_di_ (μV) recorded from Pair 1 was usually much larger than that recorded from Pair 2 [12.48 (6.33–51.93) vs. 7.32 (2.00–22.07), *p* < 0.001], while the changes of EMG_di_ before and after exercise were almost the same [139% (8%–733%) vs. 136% (1%–670%), *p* = 0.678]. The changes of EMG_di_ at different time points showed a trend of increasing first and then decreasing gradually (Figure 2). Specifically, EMG_di_ was largest in 79 participants at 3 min after exercise while 11 participants reached their maximum EMG_di_ at 5 min or longer after exercise.

The trends of heart rates in all participants during and after exercise were shown in Figure 2. The mean heart rate was 75 ± 10 (bpm) pre‐exercise, 170 ± 15 (bpm) at the mid‐exercise, and 171 ± 15 (bpm) at the end‐exercise. All participants were able to maintain the target heart rate (0.85 × (220‐age)). There was a slow decline trend in heart rate after exercise and was 112 ± 13 (bpm) at 3 min and 101 ± 12 (bpm) at 20 min after exercise.

### 3.2 Changes of EMG_di_, EMGdi/*V*_T_, and spirometer data in the control and EIB group

Sixty‐five participants were considered as the control group and 25 participants were taken as the EIB group based on changes in FEV_1_ described above (Table 1). No statistical difference in age, BMI, indoor temperature, and humidity between the two groups was observed. The prevalence of airway obstruction in the EIB group (72%) was higher than that in the control group (20%). As expected, there were significant differences in the baseline of FEV_1_ and FEV_1_/FVC (*p* < 0.001) before exercise between the two groups (Table 1). EMG_di_ in the EIB group was significantly larger than that in the control group before exercise [9.22 (2.73–18.60) μV vs. 6.92 (2.00–22.07) μV, *p* < 0.01], while there was no difference between groups before exercise in *V*_T_ [9.87 (5.01–16.44) mL/kg vs. 9.17 (2.96–20.87) mL/kg, *p* = 0.155) and EMG_di_/*V*_T_ [1.00 (0.28–2.09) vs. 0.81 (0.18–2.52), *p* = 0.082)] (Table 2).

**TABLE 2:** Changes (Δ%) in FVC, FEV_1_, EMG_di_, *V*_T_, and EMG_di_/*V*_T_ within group and the difference (*p* value) between groups.

	Control group			EIB group			*p* value
	Pre‐exercise	Post‐exercise	Δ%	Pre‐exercise	Post‐exercise	Δ%	
FVC (L)	3.60 (1.63 ~ 6.34)	3.50 (1.62 ~ 5.64)	−2 (−28 ~ 13)	3.09 (1.78 ~ 4.92)	2.75 (1.18 ~ 4.89)	−12 (−48 ~ 31)	<0.001
FEV_1_ (L)	2.79 (1.12 ~ 5.04)	2.73 (1.07 ~ 4.94)	−2 (−9 ~ 10)	1.94 (0.86 ~ 3.83)	1.46 (0.61 ~ 3.37)	−25 (−62 ~ −10)	<0.001
*V*_T_ (mL/kg)	9.17 (2.96 ~ 20.87)	16.49 (7.8 ~ 28.82)	97 (4 ~ 378)	9.87 (5.01 ~ 16.44)	15.40 (9.20 ~ 21.84)	63 (3 ~ 160)	<0.001
EMG_di_ (μV)	6.92 (2.00 ~ 22.07)	13.07 (3.00 ~ 30.33)	101 (1 ~ 417)	9.22 (2.73 ~ 18.60)	28.22 (8.80 ~ 68.73)	225 (23 ~ 670)	<0.001
EMG_di_ /*V*_T_ (μV∙kg/mL)	0.81 (0.18 ~ 2.52)	0.93 (0.17 ~ 2.02)	21 (−39 ~ 134)	1.00 (0.28 ~ 2.09)	2.04 (0.53 ~ 4.46)	124 (19 ~ 478)	<0.001

**FIGURE 3:**
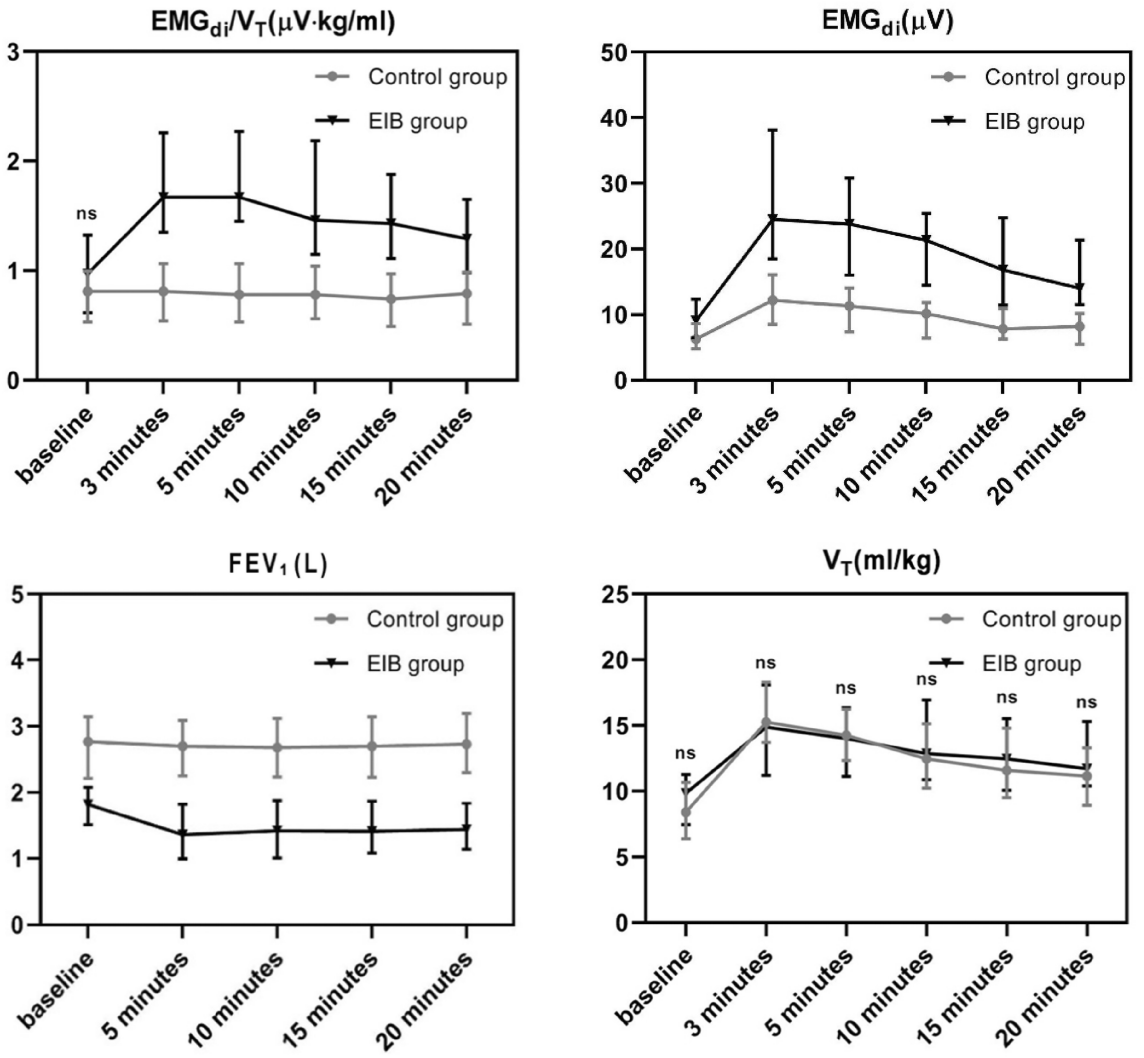
The timeline of EMG_di_ (μV), *V*_T_ (mL/kg), EMG_di_/*V*_T_ (μV∙kg/mL), and FEV_1_ (L) at various intervals of the exercise challenge test in two groups. The plots are presented by the median with interquartile range. “ns” represented no difference between the two groups.

**FIGURE 4:**
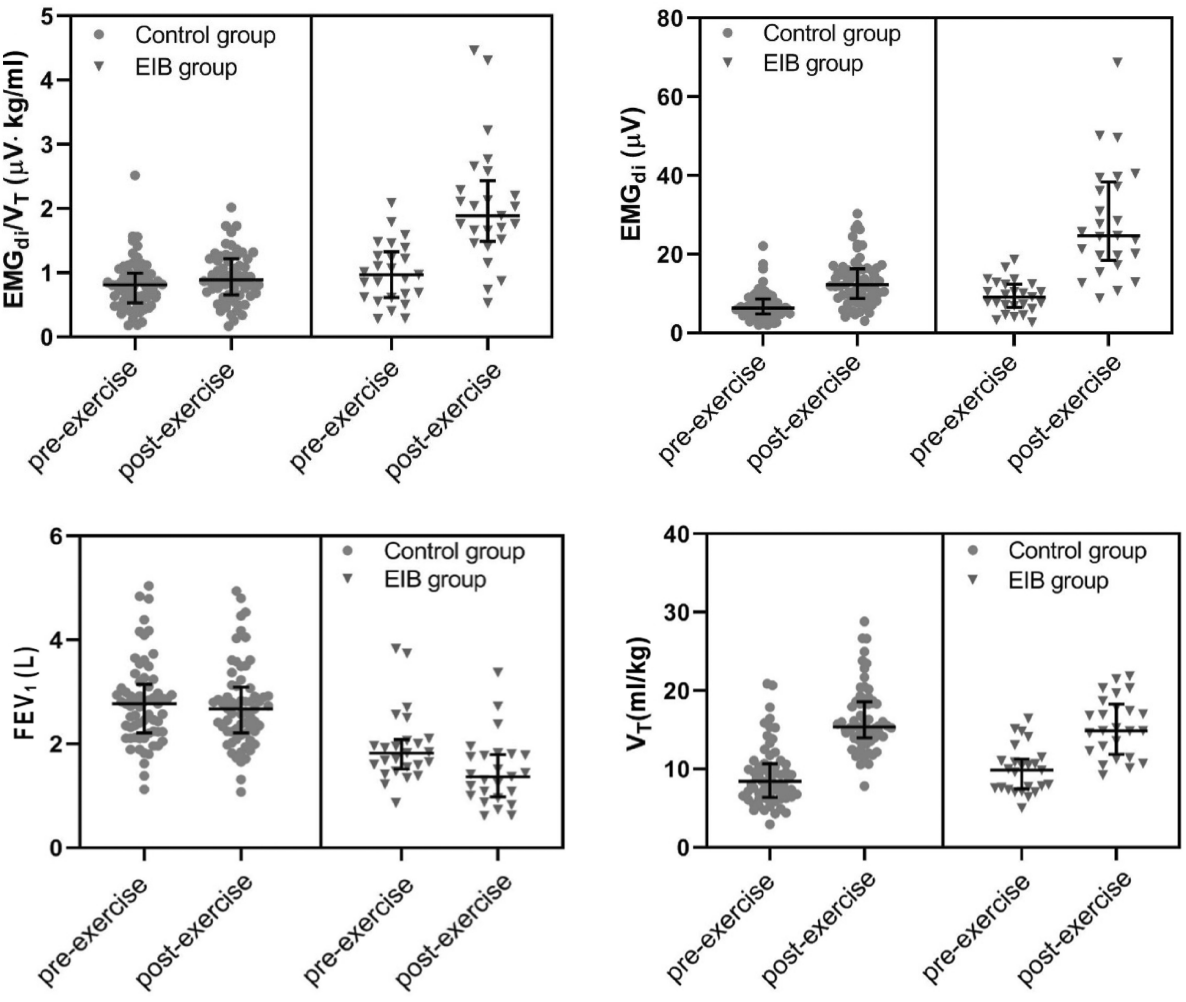
The changes of EMG_di_/*V*_T_ (μV∙kg/mL), EMG_di_ (μV), FEV_1_ (L), and *V*_T_ (mL/kg) between two different groups before and after exercise. No statistical difference was shown in the EMG_di_/*V*_T_ (μV∙kg/mL) and *V*_T_ (mL/kg) between two groups before exercise. The error bar length is the interquartile range.

EMG_di_ increased after exercise in both EIB and control groups, but the changes in the EIB group were much higher than that in the control group [225% (23%–670%) vs. 101% (1%–417%), *p* < 0.001]. Changes in EMG_di_/*V*_T_ after exercise were even larger than those in EMG_di_. Changes in EMG_di_/*V*_T_ after exercise in the EIB group were almost 6 times higher than that in the control group [124% (19% ~ 478%) vs. 21% (−39% ~ 134%), *p* < 0.001]. There were statistically significant differences in the mean of changes in *V*_T_ between the EIB group and the control group (63% vs. 97%). However, there was substantial overlap in *V*_T_ between the two groups (3%–160% vs. 4%–378%) (Table 2; Figures 3 and 4).

### 3.3 Correlation between FEV1 and EMG_di_ and ROC analysis

**FIGURE 5:**
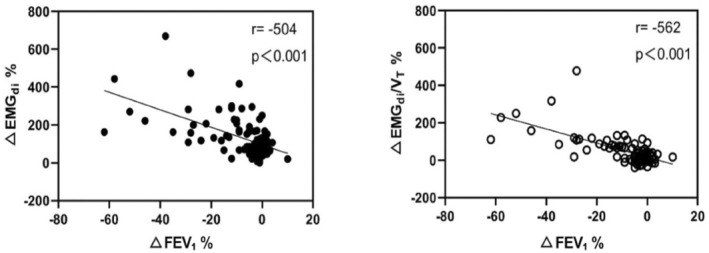
The correlation between the changes of FEV_1_, EMG_di,_ and EMG_di_/*V*_T_ before and after exercise. ΔEMG_di_% = (EMG_di_ post − EMG_di_ pre)/EMG_di_ pre × 100%; ΔEMG_di_/*V*_T_% = (EMG_di_/*V*_T_ post − EMG_di_/*V*_T_ pre)/EMG_di_/*V*_T_ pre × 100%; ΔFEV_1_% = (FEV_1_post − FEV_1_pre)/FEV_1_pre × 100%.

**FIGURE 6:**
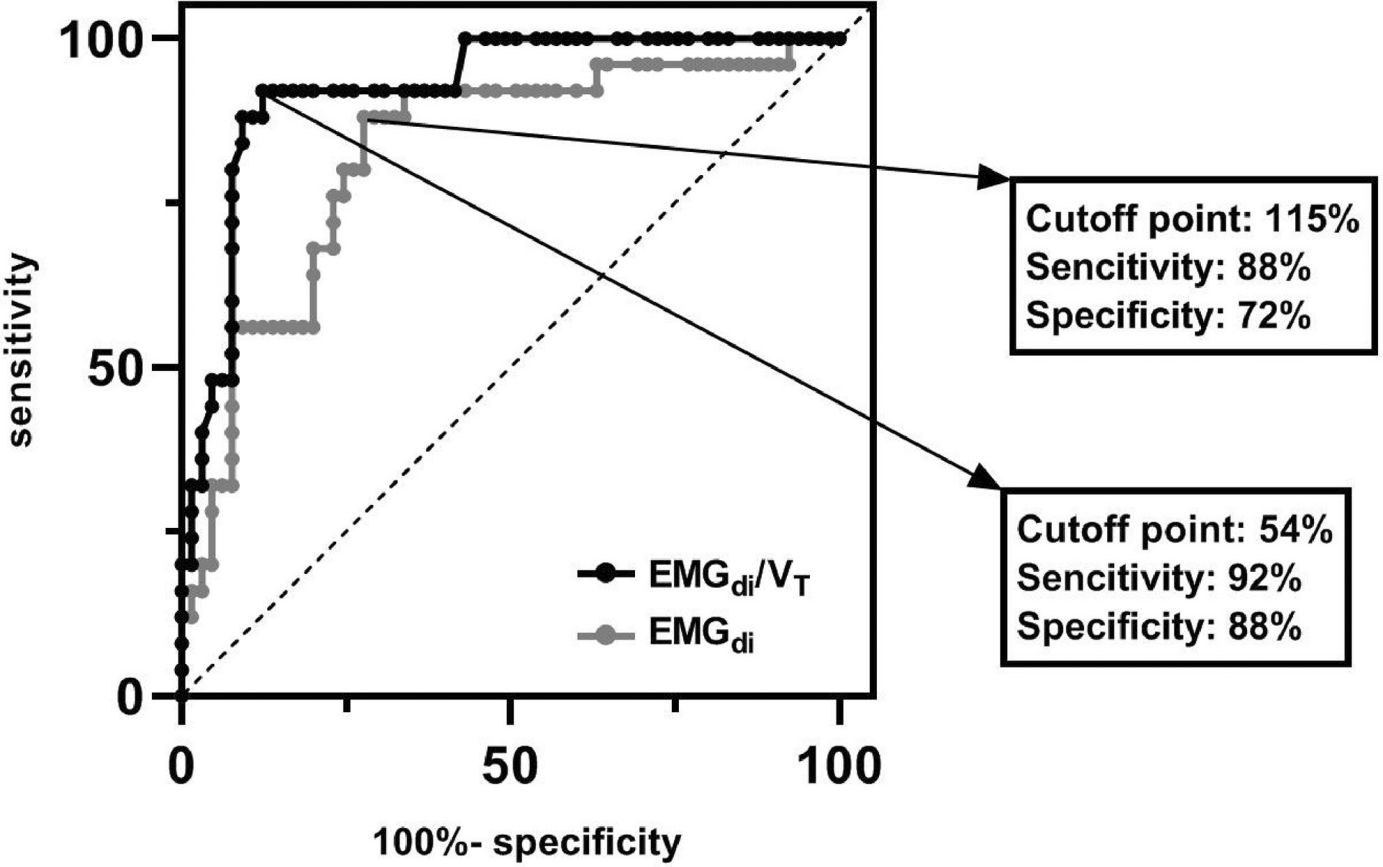
Comparison between ROC curves of ΔEMG_di_% and ΔEMG_di_/*V*_T_%. The area under curve (AUC) of EMG_di_ is 0.824 and is significantly different from the 0.922 AUC of EMG_di_ /*V*_T_ with *p* = 0.0075.

There was a moderate correlation between ΔFEV_1_% and ΔEMG_di_% (*r* = 0.504, *p* < 0.001) and between ΔFEV_1_% and ΔEMG_di_/*V*_T_% (*r* = 0.562, *p* < 0.001) (Figure 5). The ROC curves of ΔEMG_di_% and ΔEMG_di_/*V*_T_% were shown in Figure 6. The optimal cutoff point was 115% with an area under the curve (AUC) 0.82, sensitivity 88%, and specificity 72% for EMG_di_ and was 54%, with an AUC 0.92, sensitivity 92%, and specificity 88% for EMG_di_/*V*_T_. The AUC in EMG_di_/*V*_T_ was significantly larger than that in EMG_di_.

## 4 DISCUSSION

The study showed that EMG_di_ could be recorded by surface electrodes in the majority of participants during an exercise challenge test. Both EMG_di_ and EMG_di_/*V*_T_ could differentiate participants with EIB from those without EIB, but EMG_di_/*V*_T_ is more useful for the exercise challenge test.

Surface diaphragm EMG is rarely used for exercise tests because it could be affected by artifacts from other muscle activity especially during exercise. Moreover, EMG_di_ recorded from the chest wall surface electrode during tidal breathing at rest is usually small. To enlarge the EMG_di_ signal to facilitate the comparison of the change in EMG_di_ and EMG_di_/*V*_T_, an additional tube was deliberately used for increasing inspiratory load during recording EMG_di_ before and after exercise (He et al., 2021). We recorded the diaphragm EMG simultaneously from two pairs of electrodes (Figure 1). Although Pair 1 usually had a larger EMG amplitude, it also had a stronger ECG which reduces signal‐noise ratio even when modern technology is used. Consequently, the EMG_di_ recorded from Pair 2 was usually taken for comparison before and after exercise. We fixed breathing frequency at 15 times/min with a ratio of inspiration to expiration time 1/2 during the recording of EMG_di_. Although it seemed to complicate the study, we believe it was important because changes in breathing patterns after exercise could affect the amplitude of the diaphragm EMG. Our protocol used a pre‐recorded signal to assist breathing patterns; all subjects except one could follow the breathing frequency.

To minimize the effect of other skeletal muscle activity including ECG on diaphragm EMG and facilitate the comparison, diaphragm EMG was recorded at the same posture over the study. Because the amplitude of EMG_di_ is subject to change in ECG which increases with heartbeat, we analyzed EMG_di_ 3 min after exercise as heart rates are usually reduced slowly and close to a plateau by then (Best et al., 2014; Michael et al., 2017). The significant difference in EMG_di_ between changes in the EIB group and the control group further confirms the usefulness of the test. Five of 98 participants have been excluded from data analysis because of poor signal quality of EMG_di_ related to poor electrode contact. However, the problem occurred at the early stage of the study and the high‐quality EMG_di_ can be recorded after care was taken.

EMG_di_ could be used to assess the lower airway caliber under normal breathing without additional effort providing that other parts of respiratory mechanics are the same. However, the increased EMG_di_ related to an additional voluntary inspiratory effort is usually associated with an increase in *V*_T_. Consequently, it is not surprising that EMG_di_/*V*_T_ has a higher sensitivity and specificity than EMG_di_ alone in the diagnosis of EIB, which is similar to the study previously reported (He et al., 2021).

The histamine or methacholine induced bronchoconstriction test is the classical method to assist diagnosis of atypical asthma based on changes in FEV_1_ in some countries including China (Dejsomritrutai et al., 2006; Respiratory Allergy Group of Chinese Society of Allergy, 2019). The current study shows that exercise intensity based on heart rate could reliably induce bronchoconstriction which could be detected by changes in EMG_di_ recorded from chest wall surface electrodes. Because exercise is natural and the required exercise intensity for inducing EIB could be achieved by almost all the participants, exercise could replace histamine or methacholine to investigate bronchoconstriction and to diagnose asthma (Benckhuijsen et al., 1996; Haby et al., 1994).

Change in FEV_1_ after exercise is a gold standard for assessment of bronchoconstriction after exercise, but it requires participants to perform maximal inspiratory and expiratory effort, leading to test failure in the substantial number of participants, for example 10% in adults (Enright et al., 2011), 18.2% in elderly people (Pezzoli et al., 2003), and 32% in children (Gochicoa‐Rangel et al., 2013). Moreover, the other nonvolitional methods, such as impulse oscillometry parameters, should be used carefully when interpreting EIB due to the low sensitivity and specificity (Correia et al., 2022). In contrast, measurement of EMG_di_ or EMG_di_/*V*_T_ is effort independent and has a higher differentiate value with the need of only a simple breathing rhythm.

### 4.1 Study limitations

We recruited participants including those with suspected asthma and the subjects have not been further divided into subgroups based on sex and age, which are one of the limitations of the study. Moreover, we have to use a backup channel (Pair 1) to analyze data to minimize artifacts in signals, although the changes of EMG_di_ before and after exercise are the same between Pair 1 and Pair 2. Furthermore, the usefulness of changes in EMG_di_/*V*_T_ in distinguishing participants with EIB from those without EIB should be further tested by a prospective cohort study.

In conclusion, bronchoconstriction could be reliably induced by treadmill exercise. EMG_di_ could usually be recorded by chest wall surface electrodes before and after exercise. Changes in EMG_di_/*V*_T_ could be used to replace FEV_1_ to detect EIB and might be useful in patients who are unable to complete an FEV_1_ maneuver.

## AUTHOR CONTRIBUTIONS

*Conception and design*: Professor Yuanming Luo. *Analysis and interpretation*: Lishuang Wang and Senrui Wu; all authors contributed to drafting the manuscript for important intellectual content. Lishuang Wang, Senrui Wu, and Baiting He equally contributed to the work.

## FUNDING INFORMATION

This study was funded by Guangdong Science and Technology Program (Project Number 2016B010108011), National Natural Science Foundation of China (Grant Number: 82070090), Independent Project of State Key Laboratory of Respiratory Disease (Grant Number. SKLRD‐Z‐202011), Zhongnanshan Medical Foundation of Guangdong Province (Project Number: ZNSA 2020013), and Guangzhou Municipal Science and Technology Bureau, Zhongnanshan Medical Foundation of Guangdong Province (Project Number: 202201020547).
